# *In vitro* assessment of the influence of intravenous extension set materials on insulin aspart drug delivery

**DOI:** 10.1371/journal.pone.0201623

**Published:** 2018-08-16

**Authors:** Morgane Masse, Mickael Maton, Stéphanie Genay, Nicolas Blanchemain, Christine Barthélémy, Bertrand Décaudin, Pascal Odou

**Affiliations:** 1 Univ. Lille, EA 7365 – GRITA - Groupe de Recherche sur les formes Injectables et les Technologies Associées, Lille, France; 2 CHU Lille, Institut de Pharmacie, Lille, France; 3 Univ. Lille, CHU Lille, Inserm, U1008 – Controlled Drug Delivery Systems and Biomaterials, Lille, France; VIT University, INDIA

## Abstract

Insulin is a frequently prescribed drug in hospitals and is usually administered by syringe pumps with an extension line which can be made of various materials. Two insulin solutions were studied: an insulin analogue, Novorapid^®^ which contains insulin aspart and two phenolic preservatives (e.g. phenol and metacresol) and Umuline rapide^®^ with human insulin and metacresol as preservative. Some studies have indicated interactions between insulin, polyvinyl chloride (PVC) and polyethylene (PE). The aim of this work was to study such interactions between Novorapid^®^ or Umuline rapide^®^ and infusion extension line materials (PVC, PE and coextruded (PE/PVC)). Insulin solution at 1 IU/mL was infused at 2 mL/h over 24 hours with 16 different extension lines (8 in PVC, 3 in PE and 5 in PE/PVC). Ultra-Fast Liquid Chromatography with diode array detection (UFLC-DAD) was performed to quantify insulin (human and aspart) and preservatives (metacresol and phenol). Limited human insulin sorption was observed thirty minutes after the onset of infusion: 24.3 ± 12.9%, 3.1 ± 1.6% and 18.6 ± 10.0% for PVC, PE and PE/PVC respectively. With insulin aspart, sorption of about 5% was observed at the onset of infusion for all materials. However, there were interactions between phenol and especially metacresol with PVC, but no interactions with PE and PE/PVC. This study shows that insulin interacts with PVC, PE and PE/PVC at the onset of infusion. It also demonstrates that insulin preservatives interact with PVC, which may result in problems of insulin conservation and conformation. Some more studies are required to understand the clinical impact of the latter during infusion.

## Introduction

Several studies have already been made describing interactions between insulin and infusion lines. Most of the materials used to infuse drugs intravenously are polyvinyl chloride (PVC), polyethylene (PE) and PE/PVC (this is PVC whose inner surface is coated with a thin layer of PE). Zahid *et al*. [1] showed through a non-separative UV-spectrophotometry method that Actrapid^®^ (rapid human insulin) at 1 IU/mL was adsorbed on PE and PVC tubing. They demonstrated that insulin adsorption was influenced by flow rate, tubing composition and surface area. Hewson *et al*. [2] conducted a study on Actrapid^®^ adsorption on extension tubing in di(2-ethylhexyl) phthalate (DEHP)—plasticized PVC in a neonatal unit. They measured insulin by radioimmunoassay, and concluded that insulin was adsorbed on PVC and that this was increased by low concentrations and flow rates. These interactions can have severe consequences since drug concentrations are not totally delivered to patients [3]. The insulin formula used in clinical services consists mainly of insulin (human or modified) and of preservatives (phenol and/or metacresol). Preservative functions are essential, as they help to conserve insulin, a fragile protein, in its most stable conformation [4]. Fuloria *et al*. [5] performed a similar study with regular human insulin and extension line materials in PVC and PE. They concluded that episodes of hypoglycaemia observed in extremely low birth weight infants after several hours of insulin infusion were due to an excess of insulin after saturation of binding sites. Adsorption of insulin lispro and regular human insulin [6] has been studied with intravenous infusion sets in PVC and syringes in polypropylene. These authors also studied the effects of the absence or presence of an in-line filter on the release profile of insulin lispro by high pressure liquid chromatography. They concluded that the adsorption profile is the same for both insulins. Lispro and metacresol insulins were adsorbed after 5 hours on a PVC bag and tubing set, but only after a 24-hour infusion on beaker glass. Adsorption was influenced by a low flow rate, low concentration and the presence of an in-line filter.

Some studies have described strategies to reduce insulin sorption. To minimize sorption between insulin and extension set materials, Fuloria *et al*. [5] used tubing primed with insulin at 5 IU/mL to saturate insulin binding sites. Thus, after 1 hour at 0.2 mL/h, insulin recovery was 22% for unprimed and 42% for primed PVC tubings, whereas it was 19% and 80% for unprimed and primed PE/PVC tubes. Hewson *et al*. [2] used albumin to reduce insulin adsorption on the catheter. Although all these strategies are rarely used in hospitals, it is important for clinicians to take into account interactions between drugs and infusion line materials. Sorption studies on insulin mainly concern human insulin. To our knowledge, no study exists on insulin aspart although it is the most common insulin formula used in clinical services for continuous infusion. Moreover, interactions with PE/PVC tubings have not been sufficiently studied although this material is widely and increasingly used.

The main objective of this work was therefore to study the influence of intravenous extension set material on insulin aspart delivery (maintaining its complete pharmaceutical formula); the secondary objectives were to compare the drug delivery of insulin aspart *versus* human insulin and evaluate the interactions of preservatives with various materials.

## Materials and methods

### Products

Two commercialized insulin specialities were used: Novorapid^®^ (Novo Nordisk, La défense, France) and Umuline rapide^®^ (Lilly, Neuilly-sur-Seine, France). Novorapid^®^ consists of insulin aspart (100 IU/mL, 3.50 mg/mL), glycerol, phenol (1.5 mg/mL), metacresol (1.72 mg/mL), zinc chloride, disodium phosphate dihydrate, sodium chloride, hydrochloric acid and sodium hydroxide (to adjust pH) and water for injection [7]. Umuline rapide^®^ (100 IU/mL, 3.47 mg/mL) consists of human insulin, hydrochloric acid, water for injection, glycerol, metacresol (25 mg/mL), proteins of *Escherichia coli* and sodium hydroxide.

A specificity study was carried out to identify compounds of Novorapid^®^ and Umuline rapide^®^. Crystallized phenol (Cooper^®^, Melun, France), 99% metacresol (Sigma-Aldrich^®^, St-Louis, USA) and the United States Pharmacopeia (USP) human insulin standard reference (Sigma-Aldrich^®^, St-Louis, USA) were applied.

### Analytical method

An Ultra-Fast Liquid Chromatographic (UFLC) system (Shimadzu^®^, Noisiel, France) was used. It was equipped with a degassing DGU-20A3R unit to eliminate gases in mobile phases, two LC-20ADXR solvent delivery units (Prominence UFLCXR series), an SIL-20ACXR auto-sampler, a CTO-20AC column oven and an SPD-M20A photodiode array detector. The spectra were analyzed by Shimadzu Lab solution^®^ software.

#### Chromatographic conditions

The mobile phase was composed of buffer sulphate and acetonitrile (VWR chemicals, Fontenay-sous-Bois, France) (71:29, v/v). Buffer sulphate was made of anhydrous sodium sulphate (28 g/L, Na_2_SO4 0.2 M) (VWR chemicals, Fontenay-sous-Bois, France) diluted in ultrapure water (Purelab^®^ Elga, United Kingdom) and adjusted to pH 2.3 with orthophosphoric acid (Merck, Calais, France), an analytical method adopted from Xu *et al*. [8]. Column temperature was set at 25°C. Flow-rate was fixed at 0.8 mL/min and each run lasted 5 minutes. Separation of components was carried out on a Gemini^™^ C18 column (150 × 3 mm i.d., 5 μm) preceded by a C18 cartridge guard (Phenomenex^®^, Le Pecq-France). Injection volume was 20 μL and UV detection was performed at 214 nm.

#### Validation method

This method was validated following the guidelines proposed by Hubert *et al*. [9,10]. Validation was completed over three consecutive days by determining the following parameters: specificity, linearity, limits of detection (LOD) and quantification (LOQ). Limits were calculated as shown in Eqs (1) and (2) according to the methods [11] of the International Council for Harmonisation of Technical Requirements for Pharmaceuticals for Human Use (ICH)

$$\text{LOD}=\frac{3.3\;\times \;\text{standard}\;\text{deviation}\;\text{of}\;\text{intercept}}{\text{slope}}$$ (1)

$$\text{LOQ}=\frac{10\;\times \;\text{standard}\;\text{deviation}\;\text{of}\;\text{intercept}}{\text{slope}}$$ (2)

### Infusion studies

#### Preparation of insulin samples

**Fig 1:**
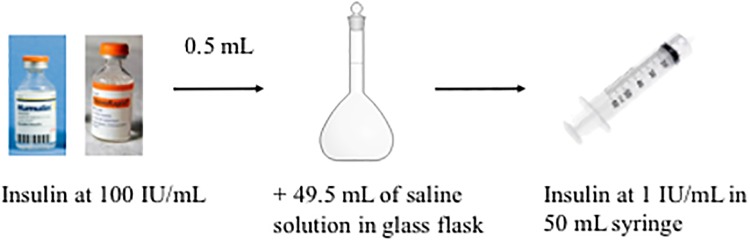
Preparation of insulin samples.

Insulin (Novorapid^®^ and Umuline rapide^®^ 100 IU/mL) was diluted in saline solution (Baxter, Guyancourt, France) to reach 1 IU/mL in 50 mL Plastipak^™^ syringes (Becton Dickinson, Le Pont de Claix, France) (Fig 1). Solutions were infused by syringe pumps (Fresenius Vial, Brézins, France) with an infusion rate fixed at 2 mL/hour over 24 hours at room temperature. The distal egress of syringes was connected to different extension tubings. No purge of the infusion lines was carried out so as not to saturate material binding sites. UFLC assays were performed at T0, T30 minutes, T1h, T2h, T3h, T4h, T5h, T6h, T7h, T8h and T24 h.

#### Materials

Table 1 summarizes the characteristics (material, internal diameter and length) of the extension tubings used. The variety of extension sets studied reflects the variety of practices observed in clinical services and some standardization would obviously be desirable, although different extension lines may be used depending on clinical practices and patient characteristics.

**Table 1:** Characteristics of medical devices.

Material	Internal diameter (mm)	Length (cm) [batch number]			Internal area surface (cm^2^)			Supplier
PVC 1,2cyclohexane dicarboxylic acid, diisononyl ester (DINCH)- plasticized	1.0	100 [PN3110]	200 [PN3120]		31.416	62.832		Cair LGL (Lissieu, France)
	1.5	100 [PN3210]	150 [PN3215]	200 [PN3220]	47.124	70.686	94.248	
	2.5	100 [PN3310]	150 [PN3315]	200 [PN3320]	78.540	117.810	157.080	
PE	1.0	100 [PE1155.10]	150 [PE1155.15]	200 [PE1155.20]	31.416	47.124	62.832	Vygon (Ecouen, France)
PE/PVC	1.0	100 [PB3110]	150 [PB3115]		31.416	47.124		Cair LGL
	2.5	100 [PB3310]	150 [PB3315]	200 [PB3320]	78.540	117.810	157.080	

PVC: polyvinyl chloride, PE: polyethylene.

#### Expression of results

Each extension line was tested in triplicate with each of the two drugs: Novorapid^®^ and Umuline rapide^®^. Solutions were collected from the extension tubings and the compounds were dosed by UFLC-DAD.

Sorption of Novorapid^®^ and Umuline rapide^®^ compounds was compared with extension tubings of the same characteristics (internal diameter = 1mm, length = 100 cm) in PVC, PE and PE/PVC by the area under the curve (AUC).

The recovery percentage of each compound was measured during the 24-hour infusion. The percentage was calculated as described in Eq 3 and related to the contact area so that variations due to the use of different lengths and internal diameters were toned down. For each material (e.g PVC, PE and PE/PVC), average recovery percentage was calculated.

$$\text{Percentage}\;\text{recovery}\;\text{of}\;\text{product}=\frac{\text{measured}\;\text{product}\;\text{concentration}\;\text{over}\;}{\text{original}\;\text{product}\;\text{concentration}}\;\times \;100$$ (3)

The cumulative amount of insulin and preservatives over the first eight hours was measured and compared to the theoretical amount so as to identify the amount administered to patients. The amount at the end of infusion (at T24h) was also measured.

#### Statistical analysis

After a Shapiro-Wilk normality test, data were compared by nonparametric tests.

For each compound, the AUC of the three materials was compared by a Kruskall-Wallis test.

This test assessed insulin delivery with each extension set. In the presence of a significant p value (< 0.05), an analysis using the Conover and Iman method with Bonferroni correction was made to detect significant differences between couples of extension sets. A Mann-Whitney test was used to compare insulin aspart and human insulin. All of these tests and graphics were made with GraphPad, Prism 6.

## Results

### Validation method

**Fig 2:**
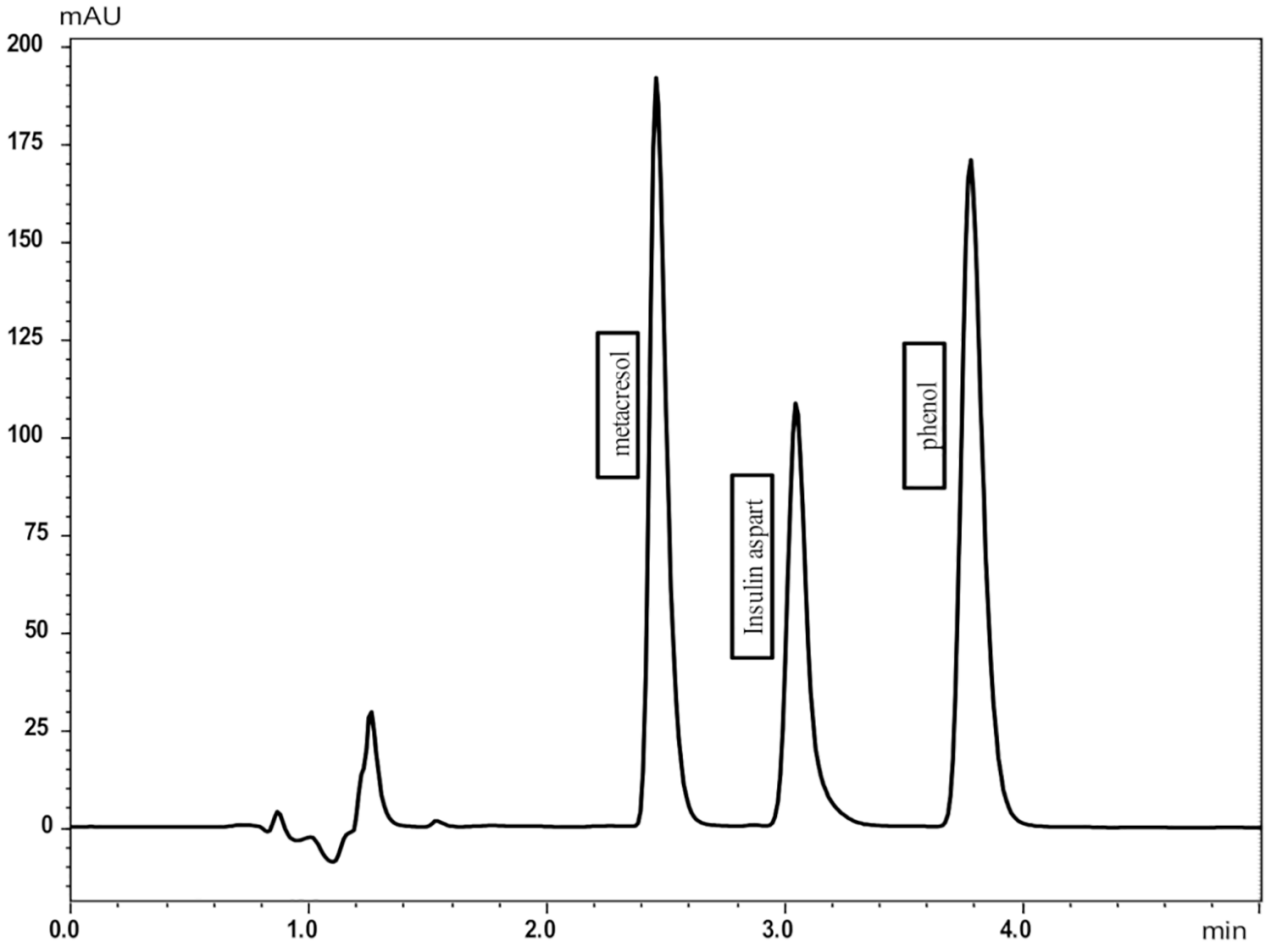
Chromatogram obtained with the UFLC-DAD method described for Novorapid^®^.

Good separation of compounds (Fig 2) was obtained with the analytical method and checked by a specificity study.

Metacresol was eluded at 2.2 min, insulin aspart at 3.0 min, human insulin at 3.4 min and phenol at 3.9 min. Good linearity and correlation were obtained for all compounds, in the ranges studied (Table 2). Relative biases were inferior to 5%. LOD and LOQ were compatible with this study.

**Table 2:** Parameters of the UFLC-DAD assay quantification.

Products	Range	r^2^	LOD	LOQ
Human insulin	0.1–2.0 IU/mL	0.9999	0.023 IU/mL	0.046 UI/mL
Insulin aspart	0.5–1.5 IU/mL	0.9999	0.029 IU/mL	0.058 IU/mL
Phenol	7.5–22.5 μg/mL	0.9999	0.139 μg/mL	0.278 μg/mL
Metacresol	8.6–25.8 μg/mL	0.9999	0.211 μg/mL	0.422 μg/mL

LOD: limit of detection, LOQ: limit of quantification

### Comparison of the area under the curve

**Fig 3:**
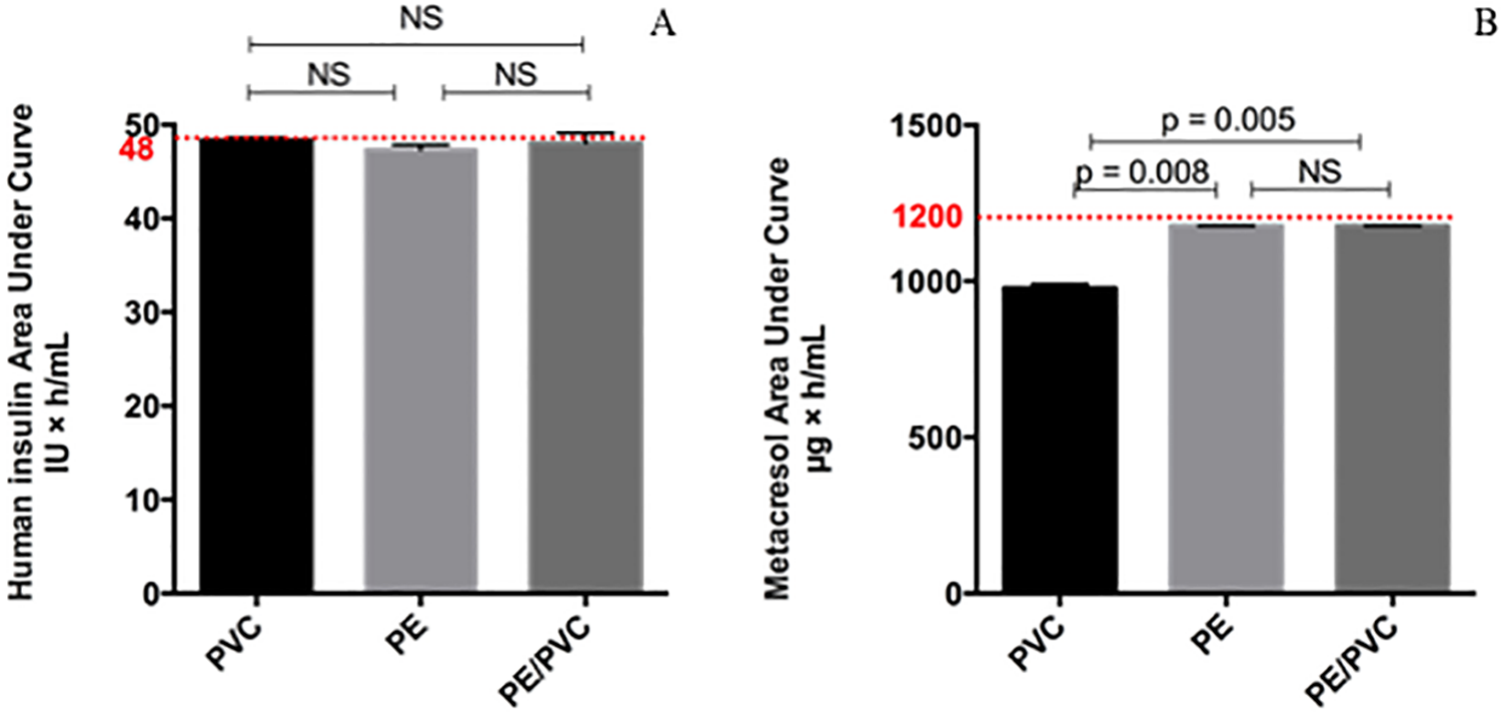
Area under the curve for Umuline rapide^®^: Human insulin (A) and metacresol (B). Statistical difference (p = 0.05), NS = not significant.

A comparison of the AUC of compounds for the extension lines (internal diameter = 1mm, length = 100 cm) was carried out for each material. For human insulin, the AUC was not significantly different from one material to another (Fig 3A) (p. Bonferroni = 0.0083). For metacresol, the AUC differed more with PVC (978.5 ± 12.4 μg×h/mL) than with PE (1176.3 ± 0.6 μg×h/mL), PE/PVC (1176.4 ± 0.8 μg×h/mL) and the theoretical value (1200.0 μg×h/mL) (Fig 3B). The percentage loss was equal to 18.5% for PVC and 2% for PE and PE/PVC.

**Fig 4:**
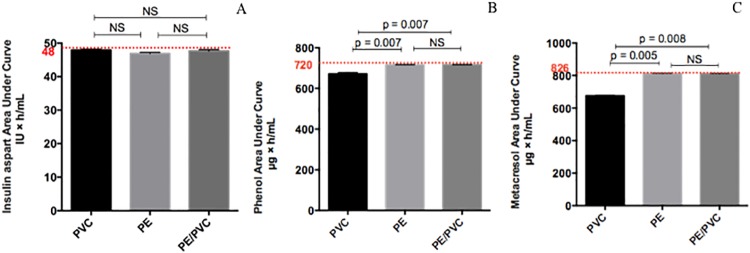
Area under curve for Novorapid^®^: Insulin aspart (A), phenol (B) and metacresol (C). Statistical difference (p = 0.05), NS = not significant.

As for human insulin, the AUC of insulin aspart was not statically different between materials (Fig 4A). For phenol and metacresol (Fig 4B and 4C), the AUC was statistically different with PVC (672.4 ± 6.6 μg×h/mL and 676.1 ± 2.4 μg×h/mL respectively) compared to PE (716.2 ± 0.7 μg×h/mL and 811.2 ± 2.4 μg×h/mL respectively), PE/PVC (716.3 ± 0.8 μg h/mL and 811.0 ± 2.1 μg×h/mL respectively) and the theoretical value (720.0 μg×h/mL and 826.0 μg×h/mL respectively). For phenol, the percentage loss was equal to 6.6% for PVC and 0.5% for PE and PE/PVC; for metacresol, the percentage loss was 18.1% for PVC and 1.8% for PE and PE/PVC.

### Recovery of compounds over 24 hours of infusion

#### Umuline rapide^®^

**Fig 5:**
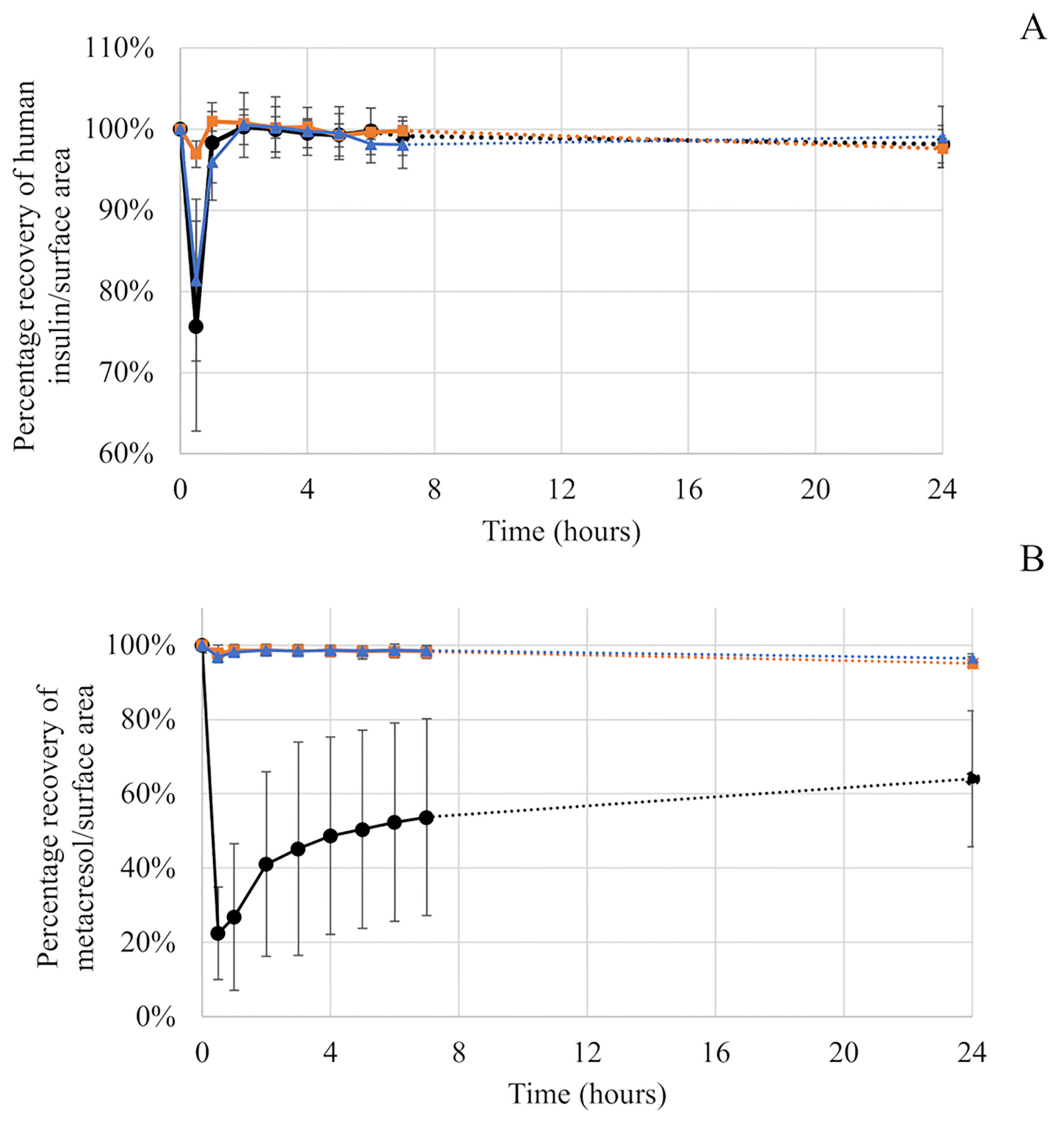
Percentage recovery for Umuline rapide^®^: Human insulin (A) and metacresol (B) during a 24-hour infusion with infusion lines in PVC, PE and PE/PVC.

Sorption of human insulin was observed thirty minutes after the onset of infusion for extension lines made of PVC (24.3 ± 12.9%) and PE/PVC (18.6 ± 10.0%), but only slight sorption (3.1 ± 1.6%) was observed with PE. However, the percentage recovery of human insulin reached about 100% over the 24 hours of infusion, for all materials (Fig 5A). With PVC, at thirty minutes, only 22.5 ± 12.5% of metacresol was administered to patients. This percentage gradually increased to reach 64.0 ± 18.3% at the end of infusion. For PE and PE/PVC, recovery percentage for metacresol equalled about 100%. A small loss of metacresol was observed at the end of infusion, the percentage being 95.6 ± 1.8% and 96.6 ± 1.2% for PE and PE/PVC respectively (Fig 5B). Standard deviations with PVC were high because three lengths and three internal diameters were used.

#### Novorapid^®^

**Fig 6:**
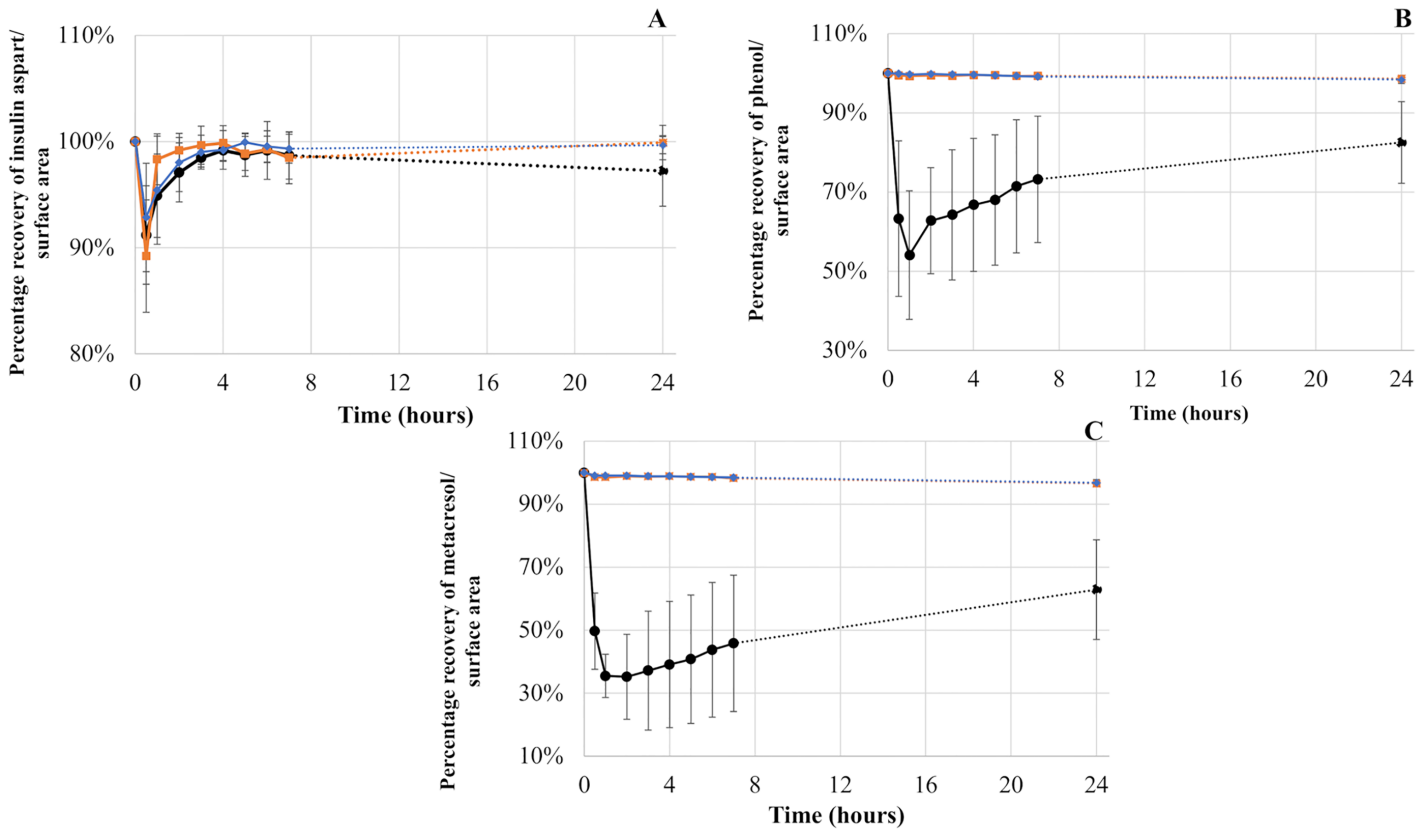
Percentage recovery for Novorapid^®^: Insulin aspart (A), phenol (B) and metacresol (C) during a 24-hour infusion with infusion lines in PVC, PE and PE/PVC.

The evolution of three different compounds of Novorapid^®^ during a 24-hour infusion is summarized in Fig 6.

The percentage of insulin aspart was nearly 100% after infusion through extension lines made of the three materials during a 24-hour infusion. However, a loss was observed after thirty minutes: the recovery percentage of insulin aspart was equal to 91.2 ± 4.6%, 89.2 ± 5.3% and 93.6 ± 7.2% for PVC, PE and PE/PVC respectively.

As for phenol, the recovery percentage remained superior to 98.6 ± 0.8% and 98.4 ± 1.0% for PE and PE/PVC respectively during the 24-hour infusion. However, for PVC, the recovery percentage was 54.1 ± 16.2% after one hour. The loss of phenol then decreased and the recovery percentage reached 82.5 ± 10.3% after 24 hours.

In the case of metacresol, the percentage remained superior to 96.8 ± 1.0% and 96.6 ± 0.5% with infusion lines in PE/PVC and PE respectively. With these two infusion lines, the lowest percentage was obtained at T24h, whereas with PVC infusion lines, the recovery percentage was equal to 35.5 ± 6.9% after 1 hour of infusion. Metacresol remained at around 40% during the first eight hours and reached 62.9 ± 15.8% at the end of the 24-hour infusion.

Standard deviations with PVC were high because several lengths and internal diameters were used and sorption results were related to contact area. In fact, eight extension sets were used.

### Cumulative amount over eight hours

#### Umuline rapide^®^

**Fig 7:**
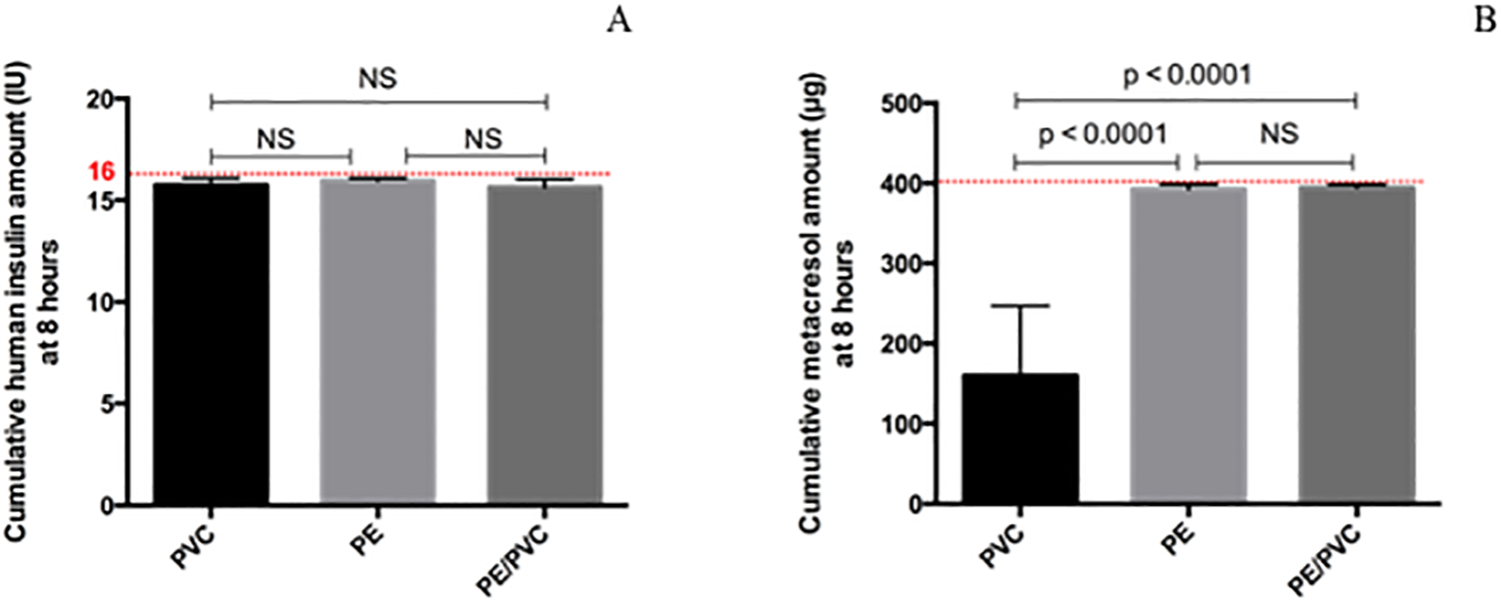
Cumulative amounts during the first eight hours of infusion for Umuline rapide^®^: Human insulin (A) and metacresol (B) with infusion lines in PVC, PE, PE/PVC. Statistical difference (p = 0.05), NS = not significant.

Cumulative amounts of human insulin after eight hours of infusion were not statistically different for the three materials nor from the theoretical amount (16 IU) (Fig 7A). The theoretical amount of metacresol over eight hours of infusion was 400.0 μg and was statistically different from the amount infused with PVC. Metacresol amount was significantly different from PVC (160.1 ± 86.9 μg) to PE (392.2 ± 6.0 μg), and from PVC to PE/PVC (394.0 ± 3.8 μg) (p. Bonferroni = 0.0083) (Fig 7B).

#### Novorapid^®^

**Fig 8:**
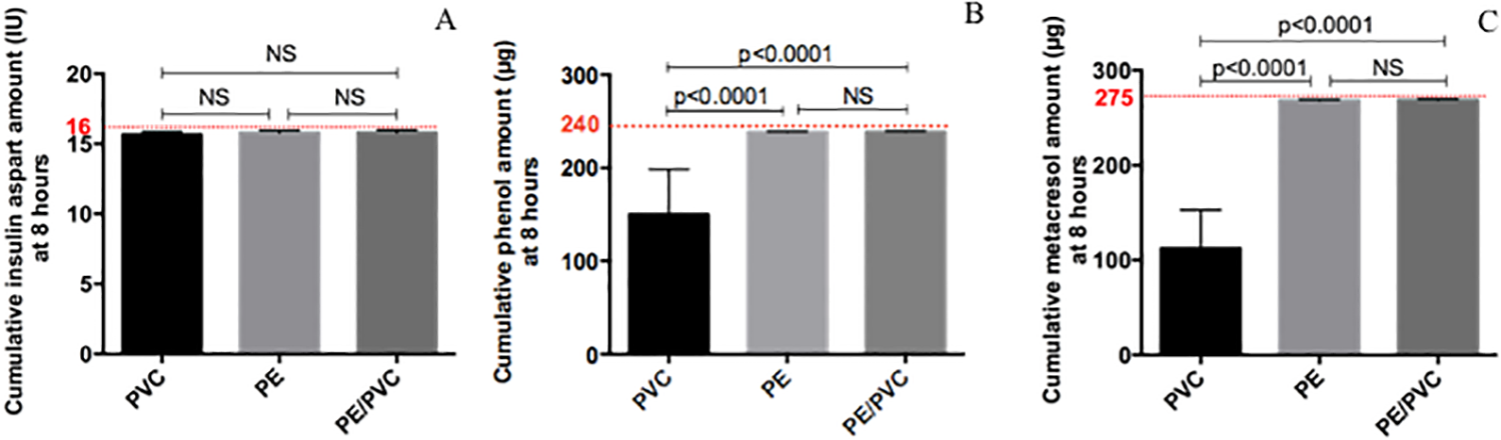
Cumulative amounts during the first eight hours of infusion for Novorapid^®^: Insulin aspart (A), phenol (B) and metacresol (C) with infusion lines in PVC, PE, PE/PVC. Statistical difference (p = 0.05), NS = not significant.

Fig 8 summarizes the cumulative amounts obtained with Novorapid^®^. Insulin amount was statistically the same with all materials, around 16 IU and equal to the theoretical amount. Phenol amount calculated after 8 hours of infusion with PVC (150.1 ± 48.5 μg) was statistically inferior to PE (238.6 ± 0.7 μg) and PE/PVC (238.1 ± 0.5 μg) and to the theoretical amount (240.0 μg).

As for metacresol, the cumulative amount after an eight-hour infusion differed statistically between PVC (112.2 ± 40.6 μg), PE (268.2 ± 0.9 μg), PE/PVC (268.6 ± 1.2 μg) and the theoretical amount (275.2 μg).

The cumulative amounts of insulin aspart, phenol and metacresol were the same for infusion lines in PE and PE/PVC (p. Bonferroni = 0.0083).

### Amount at the 24th hour of infusion

#### Umuline rapide^®^

**Fig 9:**
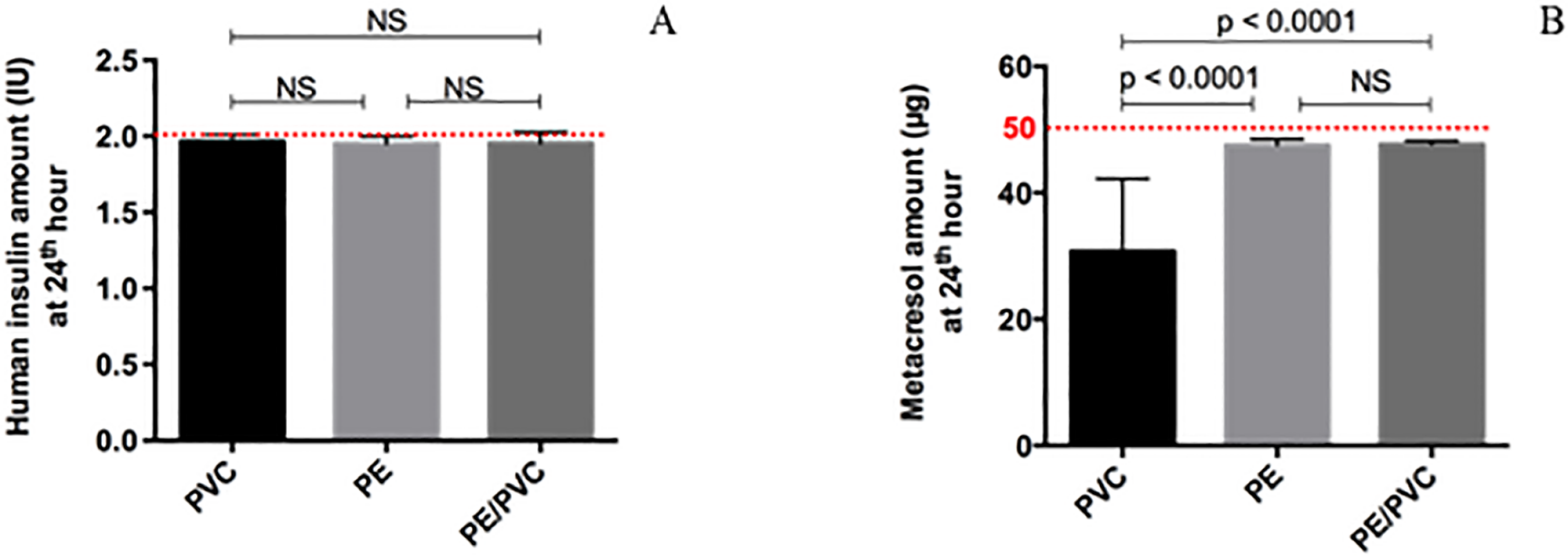
Amount at T24h for Umuline rapide^®^: Human insulin (A) and metacresol (B) with infusion lines. Statistical difference (p = 0.05), NS = not significant.

Human insulin amount was not statistically different whatever the material (2.0 ± 0.05 IU; 2.0 ± 0.05 IU; 2.0 ± 0.08 IU for PVC; PE and PE/PVC respectively) nor from the theoretical amount (2 IU). However, metacresol amount was statistically lower with PVC (30.7 ± 11.5 μg) compared to PE (47.6 ± 0.9 μg) or PE/PVC (47.7 ± 0.6 μg) (Fig 9) (p. Bonferroni = 0.0083) but was not statistically different from the theoretical amount (50 μg).

#### Novorapid^®^

**Fig 10:**
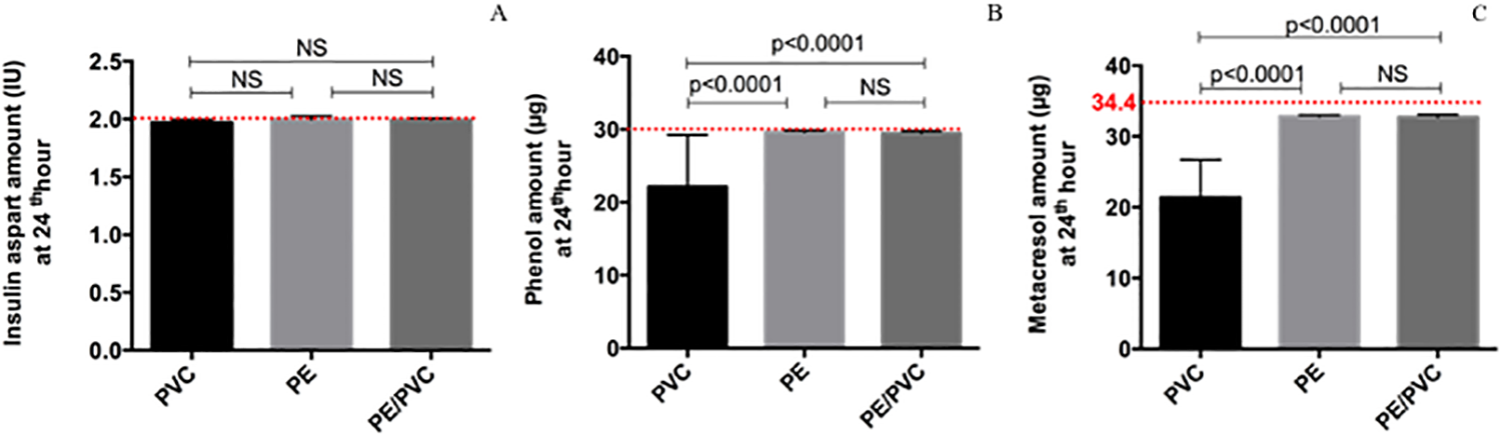
Amount at T24h for Novorapid^®^: Insulin aspart (A), phenol (B) and metacresol (C) with infusion lines. Statistical difference (p = 0.05), NS = not significant.

As for insulin aspart, the amount at T24h was the same for the three materials (1.97 ± 0.03 IU; 2.00 ± 0.03 IU; 1.99 ± 0.02 IU for PVC; PE and PE/PVC respectively). For phenol and metacresol, the amount differed statistically with PVC (22.1 ± 7.1 μg and 21.4 ± 5.4 μg respectively) compared to PE (29.6 ± 0.2 μg and 32.9 ± 0.2 μg respectively) or PE/PVC (29.4 ± 0.3 μg and 32.7 ± 0.3 μg respectively) (Fig 10) (p. Bonferroni = 0.0083).

### Insulin sorption comparison between Umuline rapide^®^ and Novorapid^®^

The loss of human insulin and insulin aspart with PVC was statistically different during the first three hours (Table 3). At thirty minutes of infusion, human insulin was more greatly adsorbed on PVC than insulin aspart (24.3 ± 12.3% and 8.8 ± 4.6% respectively).

**Table 3:** Comparison of recovery percentage of both insulins with PVC.

Time	Percentage of recovery (%) Human insulin (Umuline rapide^®^)	Percentage of recovery (%) Insulin aspart (Novorapid^®^)	P
0.5	75.7 ± 12.9	91.2 ± 4.6	< 0.0001 *
1	98.3 ± 4.9	94.9 ± 3.9	0.0003 *
2	100.3 ± 2.2	97.1 ± 2.8	0.0004 *
3	100.6 ± 2.8	98.5 ± 0.9	0.014 *
4	99.5 ± 1.8	99.2 ± 1.0	0.452
5	99.3 ± 2.6	98.7 ± 2.0	0.101
6	99.7 ± 2.9	99.2 ± 2.7	0.392
7	99.1 ± 2.4	98.7 ± 2.2	0.288
8	99.4 ± 2.4	98.6 ± 2.0	0.260
24	98.1 ± 2.3	97.2 ± 3.3	0.427

* statistical difference (p = 0.05)

At the onset of infusion with PE infusion lines, the loss of insulin aspart (10.8 ± 5.3%) was greater than that of human insulin (3.1 ± 1.6%) (p = 0.005). The recovery percentage was statistically different from one to the other during the first two hours (p = 0.013), when insulin aspart was the more adsorbed (Table 4).

**Table 4:** Comparison of recovery percentage of both insulins with PE.

Time	Percentage of recovery (%) Human insulin (Umuline rapide^®^)	Percentage of recovery (%) Insulin aspart (Novorapid^®^)	P
0.5	96.9 ± 1.6	89.2 ± 5.3	0.005*
1	100.9 ± 1.2	98.3 ± 2.4	0.013*
2	100.7 ± 1.0	99.2 ± 1.2	0.013*
3	100.2 ± 1.3	99.7 ± 1.8	0.377
4	100.2 ± 0.8	99.9 ± 1.6	1.000
5	99.2 ± 1.4	98.9 ± 1.6	0.930
6	99.6 ± 0.9	99.3 ± 1.2	0.659
7	99.8 ± 0.6	98.5 ± 2.4	0.536
8	99.6 ± 1.0	99.2 ± 1.7	0.708
24	97.6 ± 2.3	99.9 ± 1.6	0.042*

* statistical difference (p = 0.05)

For extension lines in PE/PVC (Table 5), the loss of human insulin (18.6 ± 10.0%) was statistically greater than for insulin aspart (7.2 ± 5.1%) at the onset of infusion (p = 0.001).

**Table 5:** Comparison of recovery percentage of both insulins with PE/PVC.

Time	Percentage of recovery (%) Human insulin (Umuline rapide^®^)	Percentage of recovery (%) Insulin aspart (Novorapid^®^)	P
0.5	81.4 ± 10.0	92.8 ± 5.1	0.001*
1	95.9 ± 4.7	95.4 ± 5.1	0.561
2	100.5 ± 4.0	98.0 ± 2.7	0.263
3	100.2 ± 3.8	99.0 ± 1.6	0.407
4	99.7 ± 2.9	99.2 ± 1.8	0.772
5	99.5 ± 3.3	99.9 ± 0.9	0.361
6	99.0 ± 1.4	99.5 ± 1.5	0.056
7	98.1 ± 2.9	99.3 ± 1.4	0.263
8	98.4 ± 3.2	99.4 ± 1.4	0.226
24	99.1 ± 3.8	99.7 ± 0.8	0.081

* statistical difference (p = 0.05)

## Discussion/ Conclusion

This study shows that infusion tubing materials have to be considered for drug administration and has demonstrated a close interaction between insulin and the three materials studied (PVC, PE and PE/PVC) as well as a more significant interaction between insulin preservatives and PVC. Maximum loss was observed at the onset of infusion during the first hour. With PVC however, expected preservative amounts were never administered to patients. The binding sites [5] of infusion lines were rapidly saturated with insulin but not with preservatives, especially in the case of metacresol. Few studies have so far been made on insulin interactions with PE/PVC, the use of coextruded PE/PVC lines being fairly recent. The interactions between PVC/PE or PE extension sets and insulin are therefore the same (low energy bonds). Figs 5 and 6 show that the amount of insulin delivered stabilizes at around 2 hours of infusion to reach 100%. If the dose delivered is identical to the pre-infusion dose, the interaction between PE and insulin is monolayer adsorption. Indeed, if the adsorption was a multilayer system, the dose delivered would not reach 100%.

Many care units change insulin syringes every eight hours, corresponding to staff changeover. Insulin is stable for 24h in a polypropylene syringe [12] but no rules are set down for syringe changeover. Nurses can operate following the “quick-change” protocol, or by changing both extension line and syringe. It was therefore important to assess the impact of the latter (extension line and syringe changeover) on insulin sorption.

The interaction between human insulin and insulin aspart with PVC was different during the first three hours of infusion. After thirty minutes, the loss of human insulin (24.3 ± 12.9%) was greater than that of insulin aspart (8.8 ± 4.6%) and until three hours of infusion, they were statistically different. Later, the percentage was no longer statistically different between the two insulins and reached about 100%. Thirty minutes after the onset of infusion, the sorption of insulin aspart (10.8 ± 5.3%) with PE infusion tubings was greater than that of human insulin (3.1 ± 1.6%). It was the opposite for PE/PVC, where sorption equalled 7.2 ± 5.1% and 18.6 ± 10.0% for human insulin and insulin aspart respectively.

0ur results do not in fact concord with those obtained by Zahid *et al*. [1] who found high sorption of human insulin at 1 IU/mL on PVC tubing, 200 minutes after the beginning of infusion, with an insulin percentage of around 55%. This result can be explained by a flow rate of 0.1 mL/h used for neonates, whereas in our study, flow rate was equal to 2 mL/h [13]. It may also however be due to the non-separating analytical technique used by Zahid *et al*. At its reference wavelength (210 nm), many compounds, and more particularly preservatives, are absorbed into UVs and so the analytical signals for insulin and preservatives may be mingled. Zahid *et al*. [1] also found about a 5% loss of 1 IU/mL Actrapid^®^ (human insulin) with infusion tubings in PE at 1 mL/h near the beginning (thirty minutes) of infusion. These results are also concordant with those of Hewson *et al*. [2], for whom the recovery percentage of human insulin at thirty minutes was 95.1% with extension lines in PE.

Our results however showed considerable interaction between preservatives and PVC. The two preservatives contained in insulin aspart behaved differently according to extension line materials. Indeed, with PVC, the percentage loss of metacresol (50.3 ± 12.1%) was greater than that of phenol (39.7 ± 19.6%) after thirty minutes of infusion. The same trend was observed during all infusions and so at T24h, the percentage loss was equal to 37.8 ± 15.8% and 26.3 ± 10.3% for metacresol and phenol respectively. Hence, the theoretical amount of preservatives was never administered to patients. However, these preservatives are not devoid of toxicity: Weber *et al*. [14] found that phenol and metacresol contained in insulin solutions were both cytotoxic and pro-inflammatory. They showed toxicity at 1.2 mg/mL for phenol and at 0.6 mg/mL for metacresol, indicating that metacresol is more toxic than phenol.

Standard deviations with PVC were high because several lengths and internal diameters were used and values related to the contact area. Indeed, the recovery percentage depends on internal diameter and lengths: for the same length, the larger the internal diameter, the greater the sorption; and for length, the longer it is, the greater the sorption.

The plasticizer used in PVC extension sets was identified by an analytical method previously developed [15] and can have an impact on the sorption of drug molecules. Al Salloum *et al*. [16] showed that diazepam was adsorbed more on PVC tubing plasticized with DINCH than on other tubings plasticized with DEHP, di(ethylhexyl) terephthalate and tris(2-ethylhexyl) trimellitate.

Some studies have described interactions between insulin and PE or PVC but few have used non-separative analytical methods such as UV-spectrophotometry. Actrapid^®^ is composed of human insulin and metacresol, but few studies have developed a method to dose preservatives. Moreover, no study has assessed interactions with insulin analogues.

With extension lines in PE and PE/PVC, a slight decline was observed for metacresol (around 2%) at the end of infusion, possibly due to permeation. Teska *et al*. have already noted metacresol evaporation in catheter sets after 48 hours’ incubation at 37°C [17].

The two insulin formulations did not contain the same preservatives in the same amount. Metacresol is contained in both Umuline rapide^®^ (25 μg/mL) and Novorapid^®^ (17.2 μg/mL) but the percentage loss of metacresol was greater in Umuline rapide^®^ than in Novovrapid^®^. Indeed, at T24h with PVC infusion lines, metacresol amount was 19.3 ± 11.5 μg and 12.3 ± 7.1 μg for Umuline rapide^®^ and Novorapid^®^ respectively. This can be accounted for by the fact that the insulins used did not contain the same amount of preservative and so metacresol amounts varied. Each insulin had therefore a different sorption profile with PVC, impacting differently on drug administration.

These preservative losses can have consequences on the conformational structure of insulin. Mollmann *et al*. [18] used total internal reflection fluorescence to study the adsorption of human insulin to/on Teflon particles. They concluded that insulin adsorbed to this hydrophobic material (i.e. Teflon) with high affinity. Our study showed interactions between insulin and PVC which is a hydrophobic polymer. On contact with the hydrophobic surface, insulin changes its conformation. Indeed, hexamers become monomers which can attach to each other and form filaments [19] which may occlude catheters [20]. In solution, insulin can correspond to four conformations: monomers, dimers, tetramers or hexamers [21]. In pharmaceutical formulations, insulin is presented as a hexamer, because this is the most resistant to fibrillation and degradation [22]. In the bloodstream however, hexamers dissociate to become monomers binding to insulin receptors [23]. Phenolic preservatives result in a hexameric conformation [24,25] which is the most stable insulin conformation.

Teska *et al*. [17,26] showed that insulin aspart deteriorated more rapidly during incubation at 37°C without preservatives which stabilize insulin hexamers against deamidation and intermolecular cross-linking reactions [24]. With low concentrations (2 mM) of preservatives, insulin aspart at 100 UI/mL is systematically below the monomeric size, whereas human insulin at 100 IU/mL is always eluted below the hexameric size (around 80% of hexamers) [27]. Novorapid^®^ at 100 IU/mL contains 16 mM of phenol and metacresol, Umuline rapide^®^ at 100 IU/mL contains 23 mM of metacresol. The combining propensity of insulin is a major parameter for insulin absorption after subcutaneous injection [28], indicating the importance of preservative amounts in pharmaceutical insulin formulations.

The surface morphology of the 3 materials was observed by scanning electron microscopy (SEM) before and after adsorption with novorapid^®^ (insulin aspart) at 1 IU / mL over 24 hours. No differences were noted between extension sets before and after adsorption (smooth surface of tube). In the case of extension sets after adsorption, we observed sodium salts corresponding to the diluent (NaCl 0.9%) of novorapid^®^.

Genay *et al*. [29] have already shown that an optimized infusion line can decrease the incidence of hypoglycaemic events. Similarly, effective insulin administration depends on the materials of extension tubings as well as the insulin formulation selected. Further studies are now required to assess the clinical impact of preservative loss on the biological activity of insulin.

In conclusion, this study has shown that human insulin and insulin aspart have a small interaction with PVC at the onset of infusion. No significant interaction was observed between insulin and PE or PE/PVC. However, insulin preservatives such as phenol or metacresol, were adsorbed on PVC but not on PE or PE/PVC. Thus, infusions of commercial insulin specialities should be carried out on extension sets made of PE or PE/PVC.
